# Acquired resistance to BRAF inhibition in BRAF^V600E^ mutant gliomas

**DOI:** 10.18632/oncotarget.11882

**Published:** 2016-09-07

**Authors:** Tsun-Wen Yao, Jie Zhang, Michael Prados, William A. Weiss, C. David James, Theodore Nicolaides

**Affiliations:** ^1^ Department of Pediatrics, University of California San Francisco, San Francisco, CA, USA; ^2^ Department of Neurological Surgery, University of California San Francisco, San Francisco, CA, USA; ^3^ Department of Neurology, University of California San Francisco, San Francisco, CA, USA; ^4^ Department of Neurological Surgery, Feinberg School of Medicine, Northwestern University, Chicago, IL, USA; ^5^ Northwestern Medicine Developmental Therapeutics Institute, Feinberg School of Medicine, Northwestern University, Chicago, IL, USA

**Keywords:** BRAF^V600E^, glioma, PLX4720, EGFR, Axl

## Abstract

Activating mutation of BRAF is a common finding in pediatric gliomas. As many as 14% of high grade and up to 66% of certain subtypes of low grade pediatric glioma have the BRAF^V600E^ mutation. Small molecule inhibitors that selectively target BRAF^V600E^ are FDA approved for melanoma and have shown significant efficacy in treating BRAF^V600E^ glioma in pre-clinical trials. Despite showing initial anti-tumor activity, acquired drug resistance significantly limits the benefit from being treated with BRAF^V600E^ inhibitors. Here, we have identified molecular responses to BRAF^V600E^ inhibitor treatment in human glioma models that have substantial clinical implications. Specifically, we show that BRAF^V600E^ inhibitor resistant cells upregulate pro-survival mediators such as Wnt, and additionally increase receptor tyrosine kinase activity, including EGFR and Axl, promoting resistance to BRAF^V600E^ inhibition. Our results suggest strategies to circumvent acquired resistance to BRAF^V600E^ inhibitor therapy, and thereby improve outcomes for patients with BRAF^V600E^ gliomas.

## INTRODUCTION

Oncogenic BRAF mutation occurs in approximately 8% of human cancers [1]. These include as many as 50% of metastatic melanoma, 70% of papillary thyroid cancer, 30% of ovarian cancer and 20% of colorectal cancers [1–3]. Over 90% of the BRAF mutations involve a single amino acid substitution of glutamic acid for valine at position 600 of BRAF protein, resulting in RAF kinase activation and constitutive mitogen-activated protein kinase (MAP) signaling [1]. BRAF^V600E^ mutation is found in as many as 14% of high grade and up to 66% of certain subtypes of pediatric low grade glioma. Interestingly, this mutation is much less frequent in adult glioma [4–6].

A number of BRAF^V600E^ selective small molecule inhibitors have been developed. Vemurafenib (Zelboraf) and dabrafenib (Tafinlar) are BRAF^V600E^ selective, ATP-competitive small molecule inhibitors that are FDA-approved for the treatment of melanoma [7]. We have previously shown that the tool compound vermurafenib analogue, PLX4720, reduces tumor growth and prolongs animal survival in orthotopic xenograft models of BRAF^V600E^-mutant glioma [4]. Clinical use of vemurefinib in treating glioma has produced somewhat mixed results. Robinson *et al* reported a case of complete regression in a pediatric patient treated with vemurafenib for recurrent BRAF^V600E^ giloblastoma multiforme [7]. Bautista *et al* documented partial and transient response to vemurafenib in two out of three pediatric patients with high grade BRAF^V600E^ glioma [6]. Chamberlain *et al* reported moderate single agent activity of vemurefinib against recurrent pleomorphic xanthoastrocytoma, which is a subtype of glioma with an especially high incidence of BRAF^V600E^ mutation [8]. Combined preclinical and clinical results have led to an ongoing clinical trial testing the efficacy of vemurafenib or dabrafenib for BRAF^V600E^ glioma (ClinicalTrials.gov Identifier: NCT01748149 for vemurafenib and NCT01677741 for dabrafenib).

Results from our preclinical studies using BRAF^V600E^ inhibitor monotherapy indicate that orthotopic glioma xenograft growth is delayed and/or slowed down, but not stopped, when treating mice with PLX4720, suggesting a tumor adaptive response to BRAF^V600E^ inhibitor therapy [4, 9]. In fact, acquired resistance to BRAF^V600E^ inhibitors has been observed in a number of cancer types. In melanoma, acquired resistance can be mediated through (i) upregulation of receptor tyrosine kinase (RTK) signaling, including EGFR, insulin-like growth factor 1 receptor (IGF1R) and platelet derived growth factor beta (PDGF-B) [10–12]; (ii) mutational activation of NRAS and KRAS [10, 13]; (iii) increasing COT (MAP3K8) kinase activity [14]; (iv) Raf isoform switching [11, 13]; (v) dimerization of spliced p61 BRAF^V600E^ [15]; (vi) amplification and overexpression of BRAF^V600E^ [16]; (vii) enhanced Wnt5A signaling [17, 18]; (viii) overexpression of Mcl-1 [18]; and (viiii) increased mitochondrial respiration and oxidative stress [19]. Interestingly, BRAF inhibitor insensitive melanoma regains its sensitivity after a temporary withdrawal of drug [12, 20].

We have previously shown that feedback activation of EGFR is one way in which BRAF^V600E^ glioma respond and escape from BRAF^V600E^ glioma inhibitor treatment [9]. In the present study, we show that BRAF^V600E^ inhibitor treatment additionally elevates Axl RTK activity, and also increases Wnt signaling. These tumor responses motivated our investigation of the effects from genetic as well as pharmacologic inhibition of Axl and EGFR, either concurrent with the PLX4720 tool compound treatment or subsequent to BRAF^V600E^ glioma adaptation to tool compound treatment. In each context, EGFR and Axl inhibition promoted increased tumor cell death, as well as inhibited tumor cell growth. These results support specific combination therapies for increasing BRAF^V600E^ glioma patient benefit from treatment with BRAF^V600E^ inhibitor.

## RESULTS

### Development & characterization of PLX4720-resistant BRAF^V600E^ glioma cells

**Figure 1:**
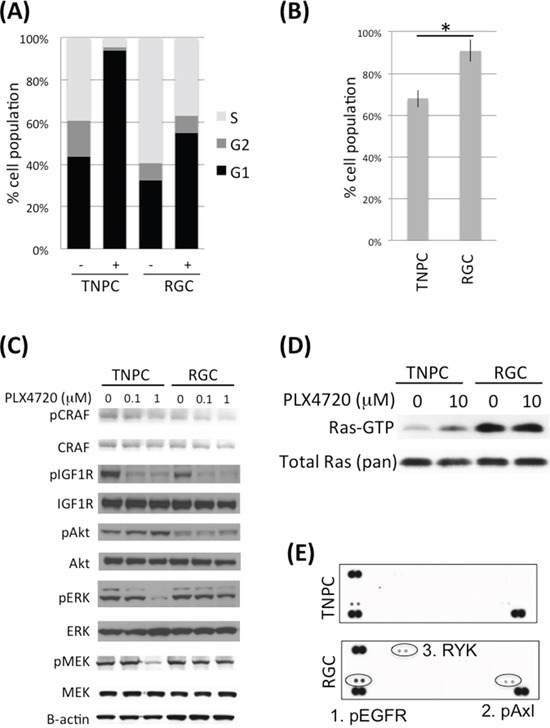
DBTRG-05MG RGC-TNPC cell pair comparison **A.** DBTRG-05MG RGCs and TNPCs were treated with 5 uM PLX4720 for 16 hours before being analyzed for cell cycle by PI incorporation; **B.** DBTRG-05MG RGCs and TNPCs were treated with 5 uM PLX4720 for 48 hours. Cell viability was measured by WST-1 assay (*p=0.001); **C.** Cells were treated with 0, 0.1 or 1 uM PLX4720 for 2 hours before being analyzed by immunoblotting using antibodies as indicated; **D.** Cells were serum starved overnight before being treated with or without 10 uM PLX4720 as indicated for 2 hours followed by stimulation with 10% FBS for 20 min. The active Ras-GTP were pulled down using GST Raf1 Ras binding domain and detected by immunoblotting; **E.** Cells were treated with 5 uM PLX4720 for 24 hours before being analyzed by human phospho-receptor tyrosine kinase arrays. Kinases differentially activated between parental and PLX4720 resistant cells are circled.

To study the mechanisms of acquired resistance, we established two human PLX4720 resistant glioma cell lines (RGCs) by culturing parental AM-38 and DBTRG-05MG cells in the presence of increasing inhibitor concentrations. RGCs that had adapted to 5 uM inhibitor showed a reduced G1 phase cell cycle population, relative to corresponding treatment naïve parental cells (TNPCs) upon initial exposure to PLX4720 (Figure 1A, Supplementary Figure S1A), and TNPC viability was more substantially impaired by PLX4720 than for corresponding RGCs (Figure 1B, Supplementary Figure S1B). RGCs were also less responsive to PLX4720 induced MAPK pathway inhibition than corresponding TNPCs, as indicated by a lesser inhibitor effect on RGC pMEK and pERK status upon PLX4720 treatment (Figure 1C; Supplementary Figure S1C).

Because increased CRAF and IGF1R activity have each been implicated in melanoma BRAF^V600E^ inhibitor resistance [11, 21], we examined RGC vs. corresponding TNPC for phospho- CRAF and IGF1R levels by immunoblotting. No appreciable differences in phospho- and total CRAF and IGF1R signals were evident upon inspection of corresponding cell pair results (Figure 1C; Supplementary Figure S1C). Nevertheless, we observed dose dependent reduction of phospho- CRAF upon PLX4720 treatment (Figure 1C).

### PLX4720 resistant glioma cells show elevated RTK and Ras activities

Elevated Ras activity has been observed in association with BRAF^V600E^ inhibitor resistance in melanoma [10]. Our comparison of Ras activity in TNPC vs. RGC, using GST Raf1 Ras pulldown assay, revealed increased Ras activity in RGC, irrespective of the presence or absence of PLX4720 in cell culture medium (Figure 1D). Interestingly, we found that PLX4720 withdrawal for 48 hours resensitized RGC to PLX4720, as indicated by pMEK analysis (Supplementary Figure S2). This finding suggests that acquired resistance to the inhibitor was maintained only as long as inhibitor treatments persisted, and that intermitted BRAF^V600E^ inhibition might be an approach to overcome drug resistance. In agreement with our previous report, TNPCs treated with PLX4720 showed increased Ras activity compared to DMSO treated control cells, most likely due to feedback activation of upstream RTK upon MAPK pathway inhibition [9] (Figure 1D).

**Figure 2:**
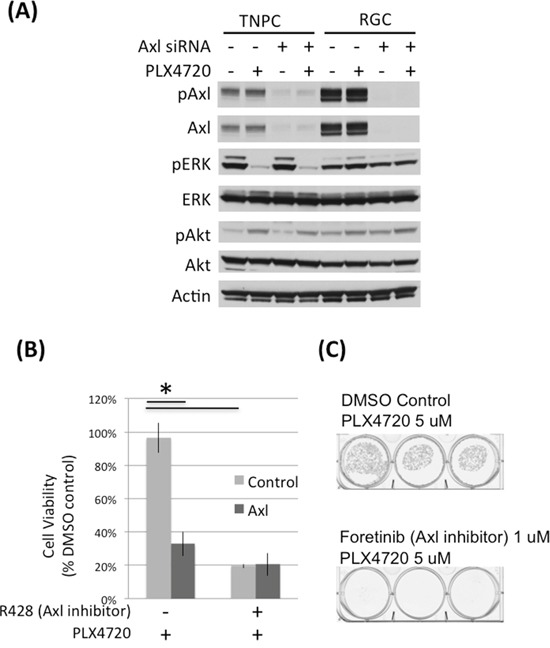
Axl knock down and inhibition impaired DBTRG-05MG RGC cell viability **A.** DBTRG-05MG RGCs and TNPCs were transfected with scramble control or Axl siRNA for 48 hours and then treated with PLX4720 for 2 hours. Molecular signaling was analyzed by immunoblotting. **B.** Control and Axl siRNA transfected cells were treated with or without 1 uM Axl inhibitor R428 for 48 hours. Cell viability was measured by WST-1 assay. (*p<0.01 for control vs Axl siRNA without R428 treatment, and DMSO vs R428 treated cells without Axl siRNA treatment) **C.** DBTRG-05MG RGCs maintained in 5 uM PLX47270 were treated with 1 uM Axl inhibitor foretinib for 3 weeks. Colonies were visualized by crystal violet staining.

We then investigated if elevated Ras activity in RGC was due to upregulation of RTK signaling by comparing RTK phosphorylation levels in RGC vs. TNPC using human phospho- RTK arrays. The results from this analysis show increased phospho Axl, EGFR and RYK in RGC (Figure 1E). Elevated Axl and EGFR activities in RGC were confirmed by immunoblotting (Figure 2A and 4A). Examination of an array of human glioma cell lines showed substantial EGFR and Axl expression in GBM cells (Supplementary Figure S3).

### Elevated Axl expression and activity are important for RGC viability

To determine the importance of Axl in conferring PLX4720 resistance property, we used genetic and pharmacological approaches to reduce Axl activity in RCG. Axl siRNA knock down and Axl inhibitors (R428 and foretinib) significantly reduced RGC cell viability determined by WST-1 (Figure 2B; Supplementary Figure S4) and colony formation assays (Figure 2C), suggesting that Axl signaling promotes the viability of PLX4720 resistant glioma cells. The viability loss conferred by Axl knock down or inhibition was profound, and additional PLX4720 exposure did not further compromise cell viability (Supplementary Figure S4).

### Exogenous Axl expression increases TNPC resistance to BRAF^V600E^ inhibitor

**Figure 3:**
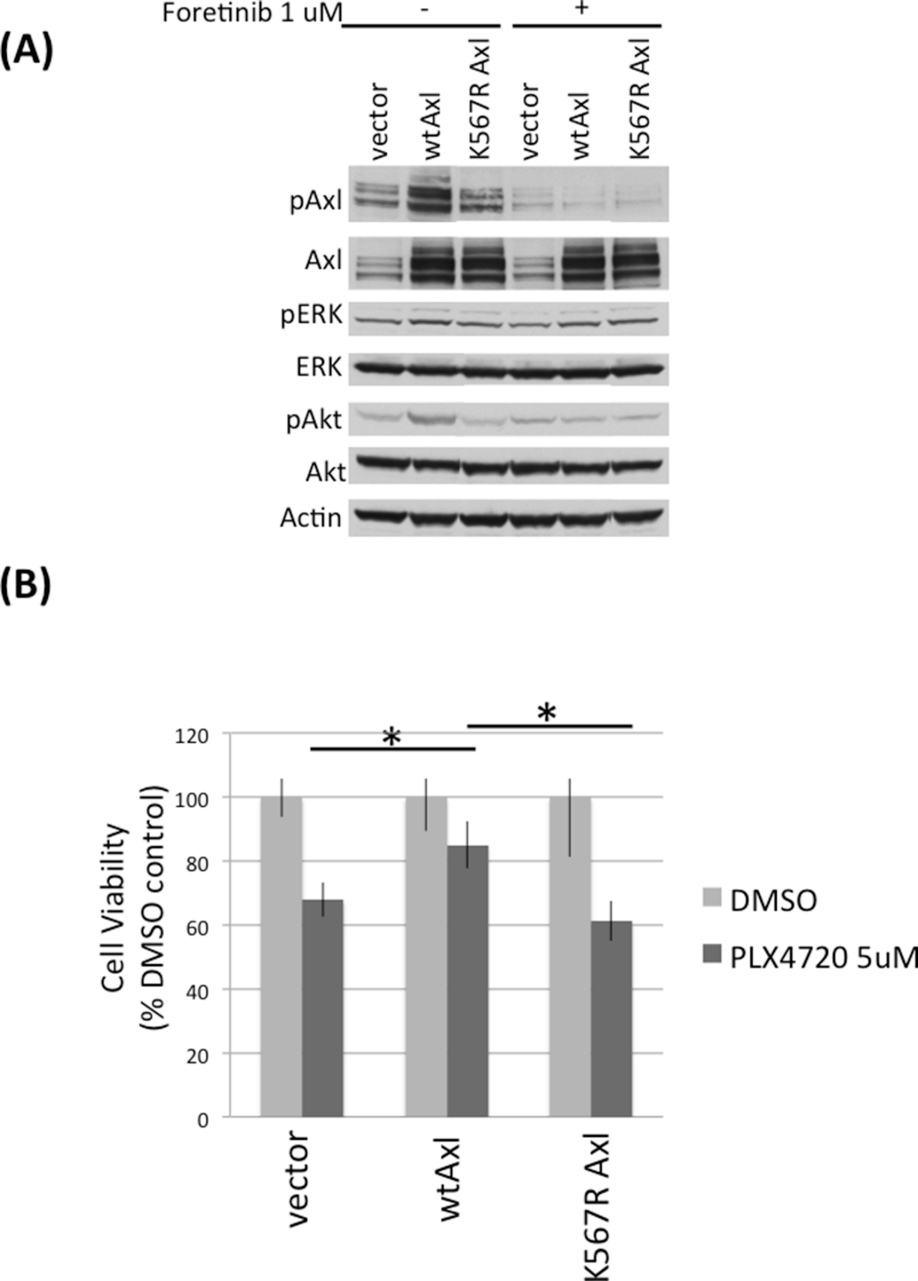
Axl overexpression protects DBTRG-05MG TNPCs from PLX4720 induced viability loss **A.** DBTRG-05MG TNPCs were transfected with vector, wt Axl, or kinase dead K567R Axl for 48 hours. Cells were serum starved overnight before being treated with 0 or 1uM foretinib for 2 hours. Cells were then stimulated with 10% FBS for 20 min to amplify signaling events before harvesting. **B.** Axl transfected DBTRG-05MG TNPCs were treated with 0.1% DMSO or 5 uM PLX4720 for 48 hours. Cell viability was measured by WST-1 assay. *p (vector vs wtAxl under PLX4720 treatment) = 0.016; *p (wt vs K567R Axl under PLX4720 treatment) = 0.028.

To investigate if Axl activity confers PLX4720 resistance, we expressed exogenous wild-type and kinase-dead K567R Axl in TNPC, then examined the response to PLX4720 treatment. TNPCs expressing exogenous wild-type Axl showed significantly higher levels of phospho-Axl compared to vector or kinase dead K567R Axl transfected TNPC (Figure 3A). Upon treatment with 5 uM PLX4720, wild-type Axl overexpressing TNPC showed significantly higher numbers of viable cells (> 80% viable cells), relative to TNPC transfected with vector (~ 70% viable cells) or kinase dead K567R Axl (~ 60% viable cells) (Figure 3B; Supplementary Figure S5A).

### Elevated EGFR expression and activity are important for RGC viability

**Figure 4:**
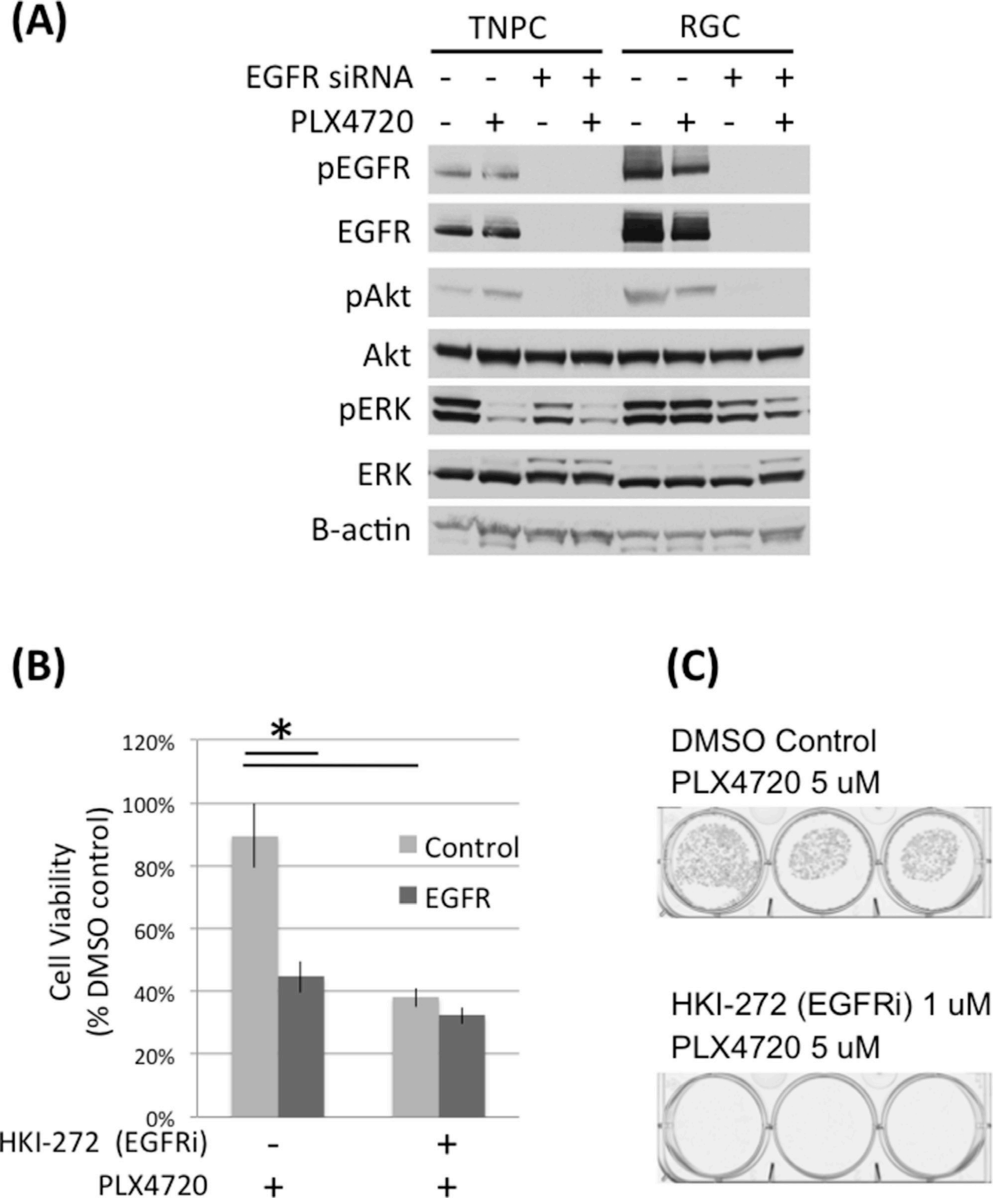
EGFR knock down and inhibition impaired DBTRG-05MG RGC cell viability **A.** DBTRG-05MG RGCs and TNPCs were transfected with scramble control or EGFR siRNA for 48 hours and then treated with PLX4720 for 2 hours. Molecular signaling was analyzed by immunoblotting. **B.** Control and EGFR siRNA transfected cells were treated with 1 uM HKI-272 for 48 hours. Cell viability was measured by WST-1 assay. (*p<0.01 for control vs EGFR siRNA without R428 treatment, and DMSO vs R428 treated cells without EGFR siRNA treatment). **C.** DBTRG-05MG RGCs maintained in 5 uM PLX4720 were treated with 1 uM HKI-272 for 3 weeks. Colonies were visualized by crystal violet staining.

To study the importance of EGFR to RGC PLX4720 resistance, we used EGFR siRNA and small molecule inhibition (HKI-272) to reduce EGFR activity in RGCs (Figure 4; Supplementary Figure S5) then examined RGC response to PLX4720. HKI-272 is an EGFR and HER2 inhibitor that demonstrated anti-tumor activity in glioma models [9]. Both genetic and pharmacologic inhibition of EGFR activity significantly reduced RGC proliferation (Figure 4B; Supplementary Figure S6) and colony formation (Figure 4C). Additionally, PLX4720 increased the efficacy of EGFR knockdown in TNGC cells but not RGCs (Supplementary Figure S6), likely due to upregulation of EGFR signaling in RGCs and therefore EGFR knockdown *per se* is more effective in reducing RGC cell viability than PLX4720, as RGC cells have bypassed PLX4720 through upstream RTK activation [9].

### Exogenous EGFR expression increases TNPC resistance to BRAF^V600E^ inhibitor

**Figure 5:**
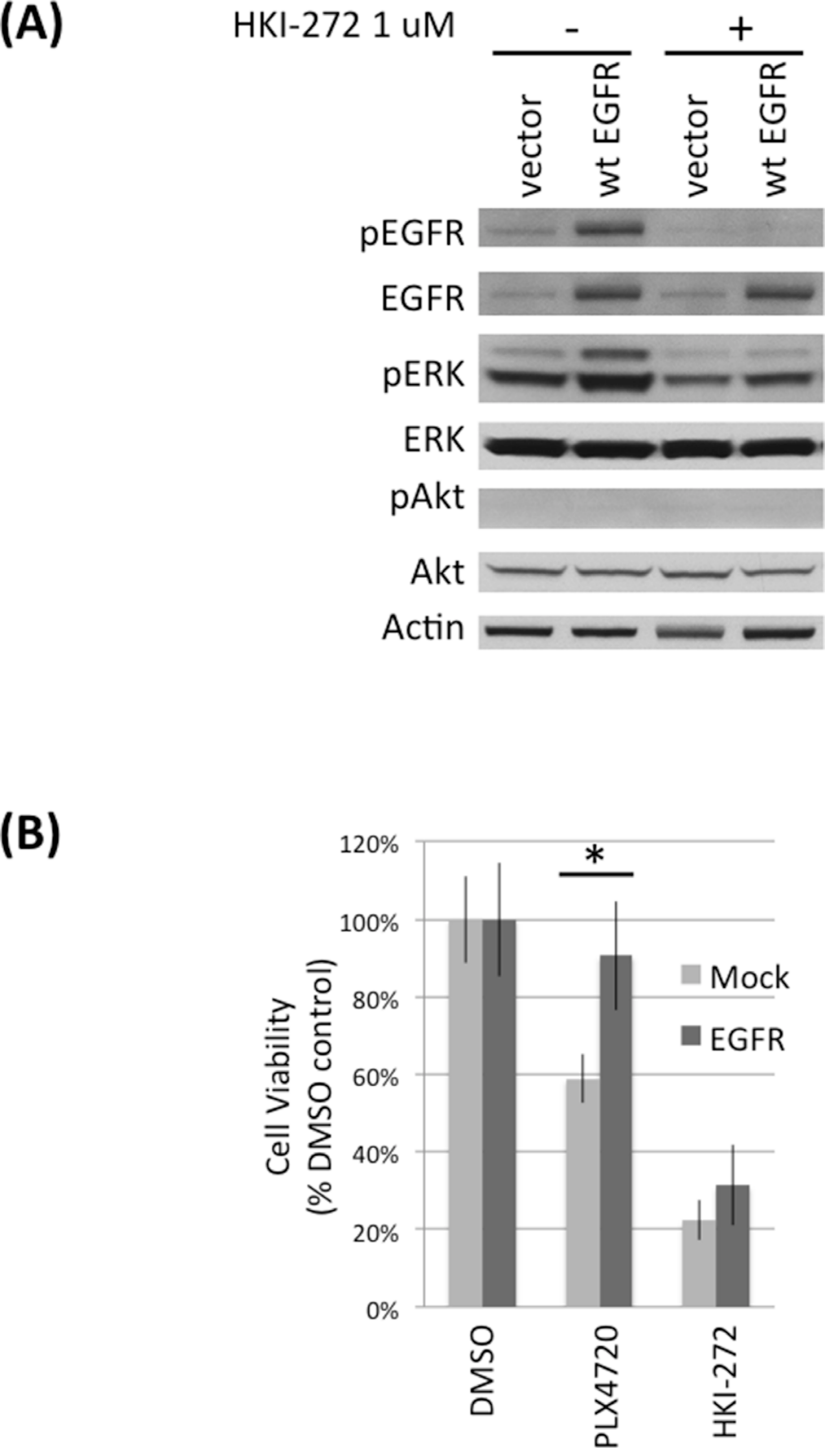
EGFR overexpression protects DBTRG-05MG TNPCs from PLX4720 induced viability lost **A.** DBTRG-05MG TNPCs were transfected with vector or EGFR for 48 hours. Cells were serum starved overnight before being treated with 0 or 1uM HKI-272 followed by 10% FBS stimulation. **B.** EGFR transfected DBTRG-05MG TNPC cells were treated with 0.1% DMSO, 5 uM PLX4720 or 1 uM HKI-272 for 48 hours. Cell viability was measured by WST-1 assay. *p (vector vs EGFR under PLX4720 treatment) = 0.001.

To determine the importance of increased EGFR activity to BRAF^V600E^ cell response to PLX4720, we expressed exogenous EGFR in TNPC (Figure 5). Significantly less PLX4720 effect was observed for TNPC expressing exogenous wild-type EGFR (~ 10% viability loss relative to vehicle-treated control cells), in comparison to mock transfected cells (~ 40% viability loss relative to control cells) (Figure 5B; Supplementary Figure S5B). HKI-272 treatment serves as negative control for EGFR activity.

### Wnt signaling is elevated in PLX4720 resistant glioma cells

We next investigated transcriptome differences between TNPCs and corresponding RGCs by gene expression arrays. Among the genes that are most differentially expressed between RGCs and TNPCs (p> 0.05), we found many involved with Wnt pathway signaling, with higher expression consistently associated with RGC (Table 1).

**Table 1:** Wnt signaling molecules overexpressed in DBTRG-05MG RGCs

Gene Name	Protein Name	Fold Change (RGC vs TNPC + DMSO)	Fold Change (RGC vs TNPC + PLX4720)
Wnt5A	Wnt5A	3.97	2.32
FZD2	Frizzled homolog 2	2.92	2.64
Dvl3	Dishevelled segment polarity protein 3	1.75	1.41
WSP1	WNT1 inducible signaling pathway protein 1; CCN4	11.3	8.07

**Figure 6:**
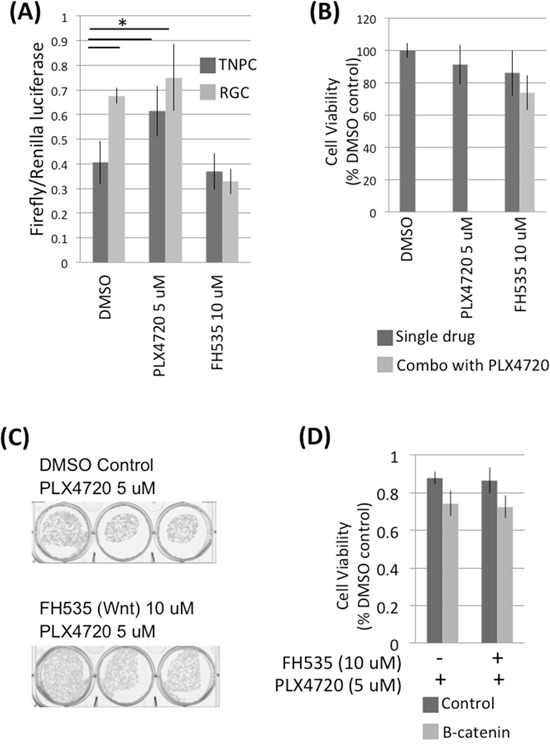
RGCs exhibit elevated Wnt signaling **A.** AM38 RGCs and TNPCs cells were treated with 0.1% DMSO, 5 uM PLX4720 or 10 uM FH535 for 24 hours. Wnt activity was measured using a Cignal TCF/LEF Reporter assay. **B.** DBTRG-05MG RGCs were treated with 0.1% DMSO, 5 uM PLX4720 or 10 uM FH535 for 48 hours. Cell viability was measured by WST-1 assay. **C.** DBTRG-05MG RGCs maintained in 5 uM PLX4720 were treated with 0 or 10 uM FH535 for 3 weeks. Colonies were visualized by crystal violet staining. **D.** DBTRG-05MG RGCs maintained in 5 uM PLX4720 were treated with 0 or 10 uM FH535 for 48 hours. Cell viability was measured by WST-1 assay. p (control vs B-catenin siRNA without FH535 treatment) = 0.046; p (control vs FH535 without B-catenin siRNA treatment) = 0.041.

We compared Wnt pathway transcriptional activity in corresponding TNPC-RGC cell pairs using a Cignal TCF/LEF reporter assay. RGC showed an almost two-fold higher level of Wnt pathway activity associated transcription compared to TNPC maintained in PLX4720 free media (Figure 6A). However, TNPC treated with PLX4720 for 24 hours showed a significant increase in Wnt signaling compared to DMSO treated control TNPC cells, indicating Wnt pathway activation occurs in just a short period of time following introduction of BRAF^V600E^ inhibitor to previously untreated cells.

### Canonical Wnt pathway inhibition does not affect RGC viability

Canonical Wnt signaling is mediated by beta-catenin, which is a transcription factor that activates Wnt responsive genes. To investigate if canonical Wnt pathway inhibition affects PLX4720 resistant glioma cell viability, we suppressed Wnt signaling by treating RGC with Wnt inhibitor FH535, and also by knocking down RCG beta-catenin via siRNA transfection (Supplementary Figure S7). 10 uM FH535 treatment did not affect RGC viability as determined by WST-1 and colony formation assays (Figure 6B and 6C). Similarly, no significant change in viability was observed in control vs beta-catenin knocked down RGCs (Figure 6D).

### Inhibition of Axl and EGFR, but not Wnt, significantly impairs RGC viability

We next examined combined pharmacologic inhibition by incubating RGCs and TNPCs with HKI-272 or foretinib or FH535, both in the presence and absence of PLX4720. HKI-272 and foretinib treatments resulted in a significant reduction in RGC and TNPC viability in both AM38 and DBTRG-05MG cells (Supplementary Figure S8, Supplementary Figure S9), however the inclusion of PLX4720 with such treatments showed little additional inhibitory effect. In contrast, we observed cell line dependent effect of FH535. FH535 treatment alone showed no significant impact TNPC cell number, whereas the combination of PLX4720 and FH535 resulted in greater growth inhibition than was observed for either drug alone in RGC of DBTRG but not AM38 cells (Supplementary Figure S8, Supplementary Figure S9).

## DISCUSSION

Vemurafenib has shown promising results in preclinical and clinical studies of BRAF^V600E^-mutant glioma [4, 22]. However, as observed with other cancer types, such as melanoma, acquired resistance to vemurafinib significantly limits the benefit from sustained BRAF^V600E^ inhibitor treatment. We have previously shown in a BRAF^V600E^ glioma model that BRAF monotherapy is inefficient in suppressing tumor growth, whereas combination with EGFR inhibitor confers significant anti-tumor efficacy, suggesting that both BRAF oncogene as well as primary resistance mechanisms have to be inhibited *in vivo* to prevent acute resistance from developing [9]. Here we provide new information regarding the molecular basis of BRAF^V600E^ glioma adaptation and acquired resistance to BRAF^V600E^ inhibition. Our results indicate an important role of Axl and EGFR signaling in conferring vemurafenib resistance in BRAF^V600E^ glioma.

We have previously shown that EGFR is hyperactivated upon BRAF^V600E^ inhibitor treatment, likely a consequence of reduced expression of the EGFR phosphatase PTPN9 upon MAPK pathway inhibition [9], and that combination EGFR and BRAF inhibition significantly improves anti-tumor efficacy in an *in vivo* model of BRAF^V600E^ glioma. Here, we show that Axl expression and activity are also increased in RGCs. Axl activity has been shown to contribute to tumor malignant phenotypes, including cell migration, survival and chemosensitivity [23–25]. As with EGFR, the increased activity of Axl is therapeutically actionable. Axl inhibitors foretinib and R428 (BGB324) have been shown by others to inhibit glioma cell proliferation, migration, collagen invasion and survival *in vitro* [26, 28]. Treatment of rodents bearing subcutaneous U251 glioma xenografts with foretinib has been shown to inhibit tumor growth and prolonged animal survival [26]. Similar results were seen in animals bearing glioma cells modified with a dominant-negative Axl expression construct [27]. Importantly, the previous studies of Axl in glioma used tumor cell sources expressing wild-type BRAF. Here we provide the first evidence showing that Axl inhibition suppresses BRAF^V600E^ glioma, and that Axl inhibition is a therapeutic strategy deserving of further development for clinical translation.

In squamous cell carcinoma (SCC) cells Axl has been shown to dimerize with EGFR and promote ligand independent phosphorylation of EGFR, leading to increased activation of phospholipase Cγ (PLC γ) and protein kinase C ζ (PKCζ), as well as mTOR [28]. Zang *et al* have shown that genetic and pharmacologic inhibition of Axl help prevent acquired resistance to EGFR inhibition, in EGFR–mutant lung cancer models [29]. In breast cancer, activated EGFR has been shown to transactivate Axl. These observations, combined with new results reported in the present study, suggest a coordinated Axl + EGFR tumor cell response in adapting to various small molecule inhibitor therapies.

We also observed increased expression of multiple Wnt pathway genes, including Wnt5A, FZD2, Dvl3 and WSP1, in RGCs, with elevated Wnt pathway-associated transcription in these cells confirmed by TCF/LEF reporter results. However, in contrast to previous report with U251 (BRAF wild-type) glioma cells [30], genetic and pharmacological inhibition of Wnt signaling did not affect BRAF^V600E^ glioma cell growth and did not sensitize RGCs to BRAF^V600E^ inhibition. Wnt 5A is an important component of the non-canonical Wnt signaling effects, which include cytoskeleton organization and function [31], potentially through frizzled receptor interaction with RYK, the expression of which we also found to be increased in RGCs (Figure 1E). Future work is required for detailed investigation of non-canonical Wnt signaling effects, and of relationships with tumor phenotypes other than cell viability.

Irrespective of the nature of Wnt pathway involvement with BRAF^V600E^ glioma cell adaptation to BRAF^V600E^ inhibitor treatment, our current work provides information important to improving the duration and/or extent of BRAF^V600E^ glioma therapy by interfering with compensatory cell responses that involve increasing Axl and EGFR activities. Given the availability of clinically approved compounds for inhibiting these RTKs, there will certainly be opportunities for translating our observations to evaluate combination therapies for treating patients with BRAF^V600E^ glioma.

## MATERIALS AND METHODS

### Cell source and investigational agents

AM38, DBTRG-05MG, LN229, NMC-G1 and U87MG cells were purchased from ATCC. 8MGBA, 42MGBA were obtained from DSMZ. ATRT lines BT12, BT16 and 794 was kindly provided by Dr N. Foreman (University of Colorado). CHLA-07-BSGBM was the provided by the courtesy of Dr Anat Erdreich-Epsten (Children's Hospital Los Angeles). SF188, SF9744, SF9841 and SF9867 were obtained from the Brain Tumor Research Center (University of California, San Francisco).

Wild-type and K567RAxl in pcDNA3.1 (+) vector was kindly provided by Dr Trever Bivona (Helen Diller Cancer Center, UCSF) [30]. pcDNA3.1-v5/His plasmids containing human full-length *EGFR* has been described previously [32]. PLX4720 was provided by Plexxikon Inc (Berkeley, CA, USA) and HKI-272 (Neratinib) was purchased from TSZ Scientific LLC (MA, USA). Axl inhibitors foretinib (GSK1363089) and R428 (BGB324) were purchased from Selleckchem (Houston, Texas, USA) and Axon Medchem (Reston, Virginia, USA) respectively. All drugs were dissolved in dimethylsulfoxide (DMSO) at 10 mM and stored at −20°C. The final DMSO concentration in all experiment was less than 0.1% in medium.

### Cell culture and transfection

All cell lines were maintained in DMEM supplemented with 10% fetal bovine serum, 1% penicillin and streptomycin, and 1% non-essential amino acid. PLX4720 resistant cells were generated by culturing parental AM38 and DBTRG-05MG cells in increasing concentrations of PLX4720 to achieve chronic selection. PLX4720 resistant cells were maintained in the presence of 5 uM PLX4720. For Axl, EGFR and B-catenin siRNA knock down experiments, cells were transfected with Dharmacon siGENOME non-target siRNA, Axl SMARTpool siRNA or EGFR SMARTpool siRNA following manufacturers instruction (Thermo Scientific, MA, USA). For Axl and EGFR overexpression, transfection was achieved using the Amaza Basic Glial Cells Nucleofector Kit (Lonza, Germany) following manufacturers instructions.

### Western blotting

Proteins were extracted from cells using cell lysis buffer (Cell Signaling Technology, Danvers, Massachusetts, USA) supplemented with proteinase (Roche, Upper Bavaria, Germany) and phosSTOP phosphatase inhibitor cocktail (Roche). Proteins were resolved by SDS-PAGE and transferred onto polyvinylidene difluoride membranes, which were then probed with primary antibodies followed by horseradish peroxidase-conjugated secondary antibody, and visualized by ECL (GE Healthcare, Buckinghamshire, UK). Antibodies against phospho-ERK, phospho--Akt, total Akt, phosphor-CRAF, total CRAF, phospho-IGF1R, total IGF1R, phospho-MEK, total MEK, phospho EGFR, Ras, phospho Axl, total B-catenin were purchased from Cell Signaling Technology. Total EGFR and total ERK antibodies were purchased from Santa Cruz Biotechnology (Dallas, Texas, USA) and total Axl was obtained from R&D Systems (Minneapolis, Minnesota, USA). Antibody specific for p-EGFR (1173) was obtained from Novus Biologicals, and antibodies specific for Beta-Tubulin was from Milipore. Anti-Wnt5A antibody was purchased from Abcam (Cambridge, Massachusetts, USA).

### Cell viability assay

Cells were seeded onto 48-well plates at 2500 to 3000 cells per well. After 16 hours of seeding, cells were maintained in drug containing culture media for 72 hours. Cell viability was determined by WST-1 assay (Roche) according to manufacturer's instructions. 450 nm absorbance was measured using a microplate reader (Gen5, BioTek), with background reading at 800 nm subtracted. Percent viability was normalized against cell treated with 0.1% DMSO as control.

### Cell cycle analysis

Cells were treated with or without drugs for 24 hours before harvesting. The trypsinized cells were washed with PBS and fixed in ice-cold 70% ethanol overnight followed by staining with propidium iodide (20 ug/ml) (Invitrogen, Carlsbad, California, USA) in PBS containing RNaseA (0.4 mg/ml) (Invitrogen). Fluorescence levels (488nm excitation) were measured by a FACSCalibur (Becton Dickinson, San Jose, California, USA), and data was analyzed using the ModFit software (Verity Software House, Topsham, Maine, USA).

### Microarray analysis

RNA was isolated using the RNeasy Mini kit (Qiagen, Valencia, California, USA) and quantified using a NanoDrop Spectrophotometer (Agilent Technologies, Santa Clara, California, USA). The NuGEN Pico V2, based on Ribo-SPIA technology, was used for amplification, fragmentation and biotin-labeling. The labeled cDNA was hybridized to Human GeneChip Gene 2.0 ST microarrays (Affymetrix, Santa Clara, CA). The signal intensity fluorescent images produced during Affymetrix GeneChip hybridizations were read using the Affymetrix Model 3000 Scanner and converted into GeneChip probe results files (CEL) using Command and Expression Console software (Affymetrix). The analysis of the microarray data was done by first normalizing for array-specific effects by Affymetrix's “Robust Multi-Array” (RMA) normalization. The normalized arrays values were then reported on a log2 scale. For statistical analyses, all array probesets where no experimental groups had an average log2 intensity greater than 3.0 were removed. This is a standard cutoff as below which expression is indistinguishable from background noise. Linear models were then fitted for each gene using the Bioconductor limma package in R [33]. Moderated t-statistics, fold-change and the associated p-values were calculated for each gene. The experiments were performed in triplicates.

## SUPPLEMENTARY FIGURES


