# Supplementary material for: Acquired resistance to BRAF inhibition in BRAFV600E mutant gliomas
Source: Oncotarget. 2016 Sep 7;8(1):583–95. doi: 10.18632/oncotarget.11882 (PMC5352180; doi:10.18632/oncotarget.11882)
Supplement: Supplementary file 1 [file oncotarget-08-583-s001.pdf]

Acquired resistance to BRAF inhibition in BRAF^{V600E} mutant gliomas

SUPPLEMENTARY FIGURES

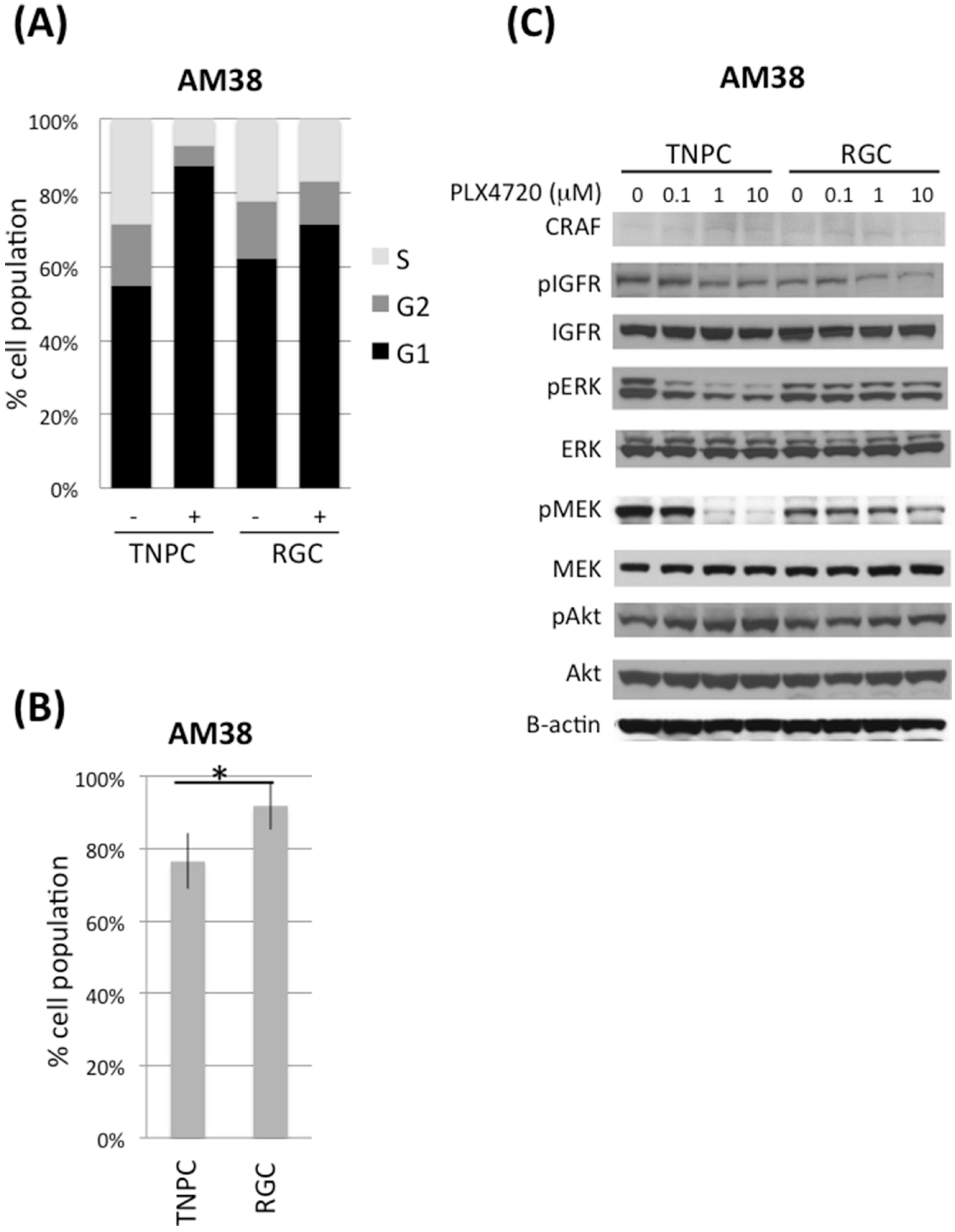

Supplementary Figure S1: AM38 RGC-TNPC cell pair analysis. A. AM38 RGCs and TNPCs were treated with 5 uM PLX4720 for 16 hours before being analyzed for cell cycle by PI incorporation; **B.** AM38 RGCs and TNPCs were treated with 5 uM PLX4720 for 48 hours. Cell viability was measured by WST-1 assay (*p=0.048); **C.** Cells were treated with 0, 0.1 or 1 uM PLX4720 for 2 hours before being analyzed by immunoblotting.

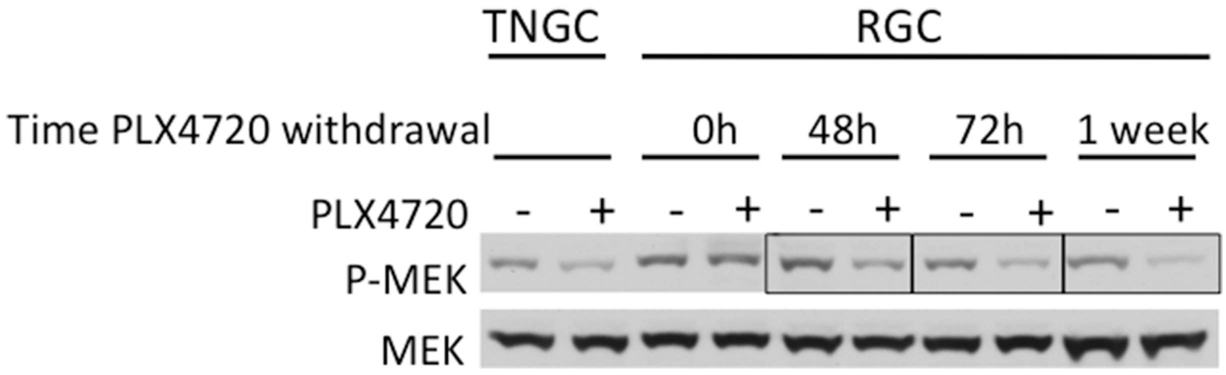

Supplementary Figure S2: DBTRG-05MG RGCs maintained in 5 uM PLX4720 were cultured in PLX4720 free media for 0, 48, 72 hours or one week. Cells were serum starved for 16 hours before being treated with 5 uM PLX4720 for 2 hours followed by 10% FBS stimulation. Molecular signaling was analyzed by immunoblotting.

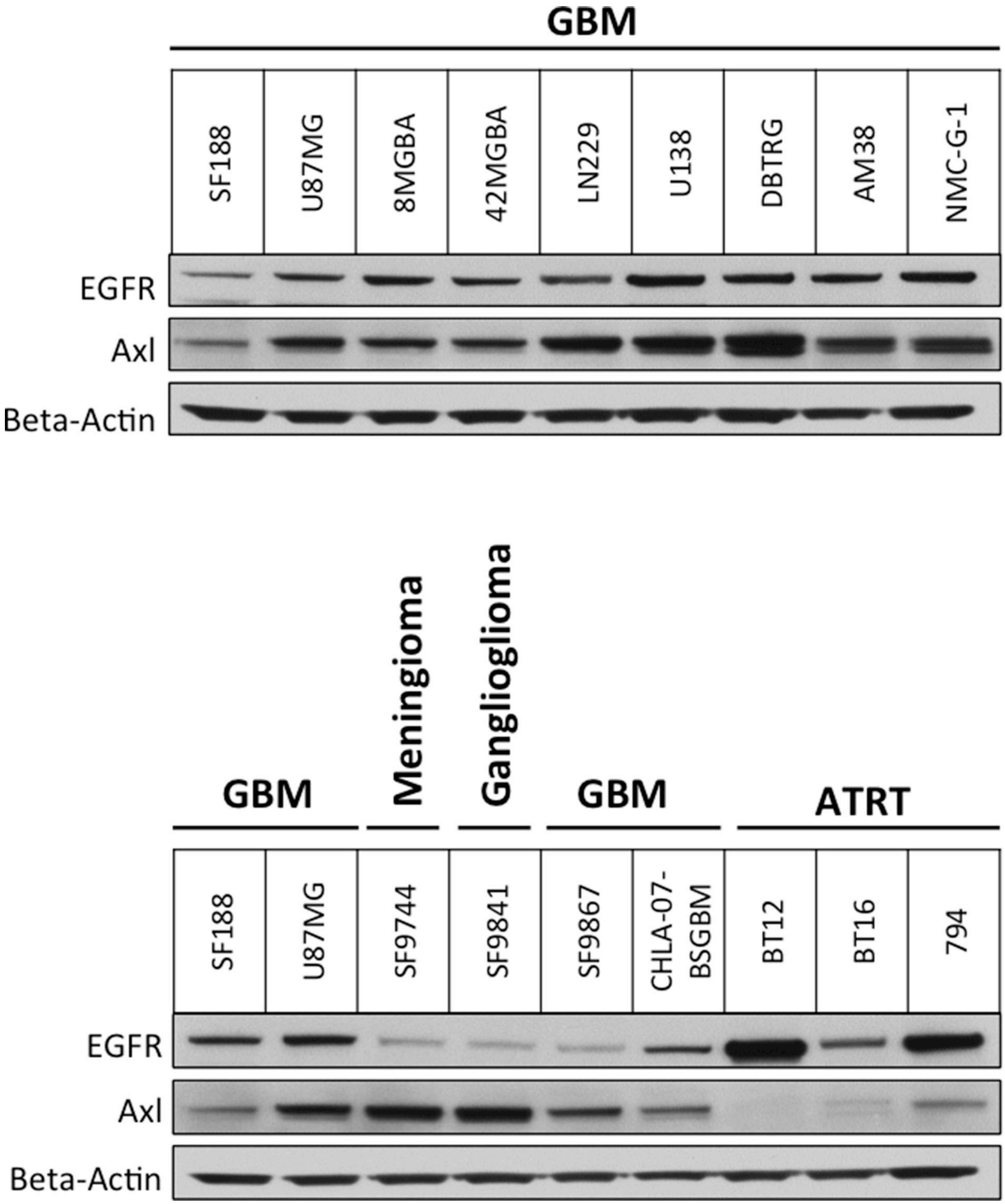

Supplementary Figure S3: Axl and EGFR are highly expressed in human glioma cell lines. 15 human glioma cell lines, including 13 GBM and 3 ATRT cells, were examined for Axl and EGFR expression by immunoblotting.

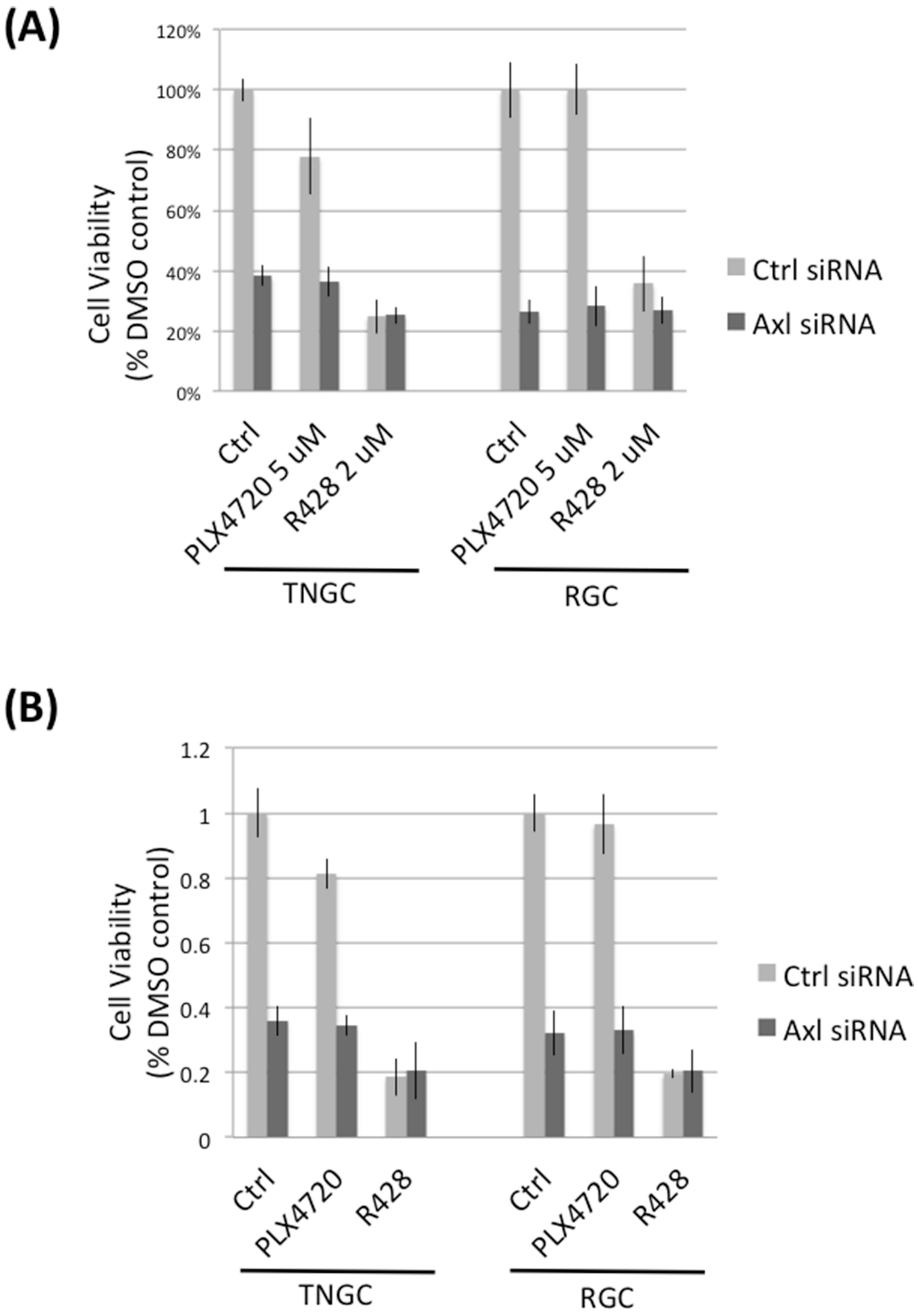

Supplementary Figure S4: AM38 A. and DBTRG-05MG B. RGCs and TNPCs were transfected with scramble control (light grey bars) or Axl siRNA (dark grey bars) and treated with 0.1% DMSO, 5 uM PLX4720 or 1 uM R428 for 48 hours. Cell viability was measured by WST-1 assay.

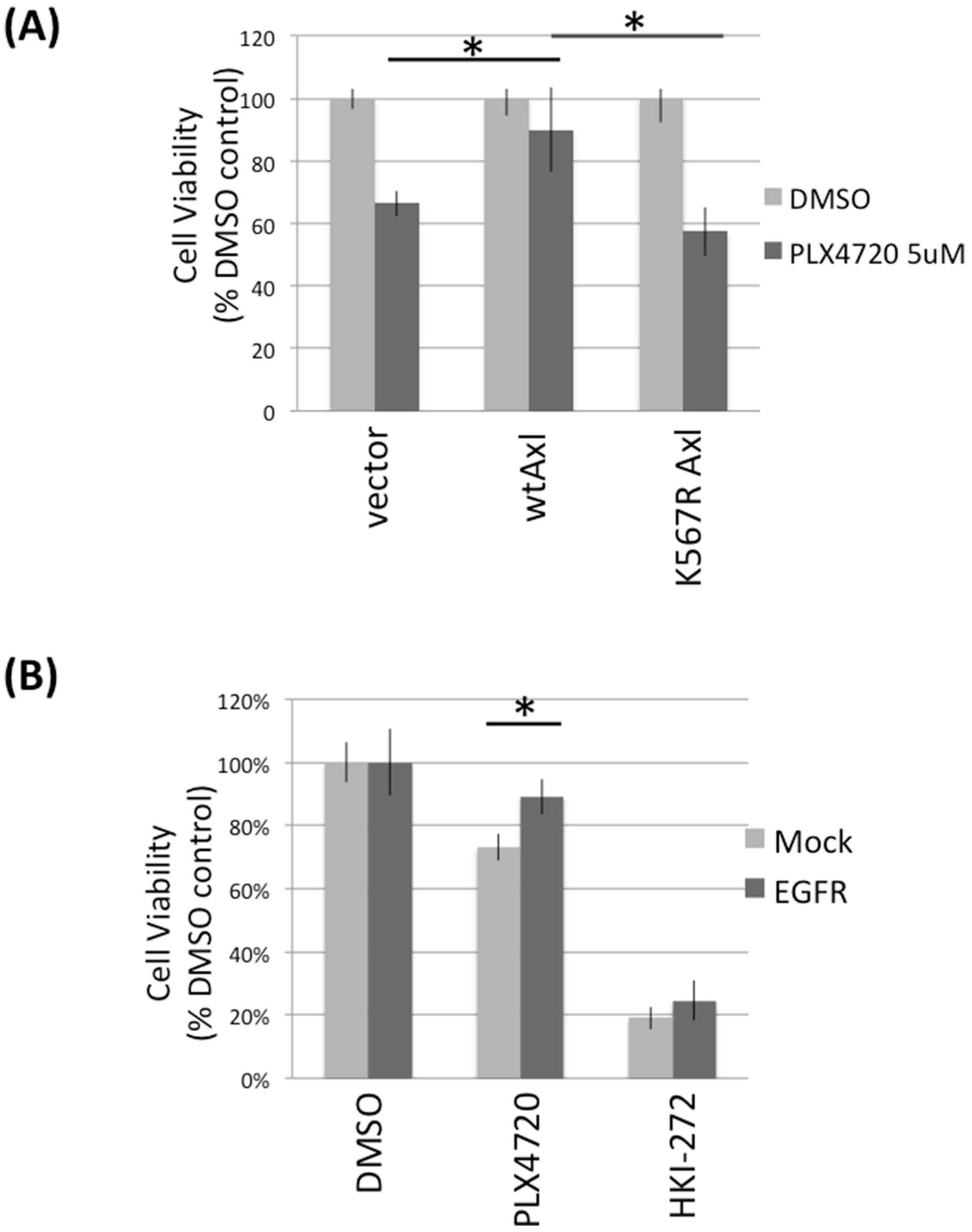

Supplementary Figure S5: Axl and EGFR overexpression protects AM38 TNPCs from PLX4720 induced viability loss. A. Axl transfected AM38 TNPCs were treated with 0.1% DMSO or 5 uM PLX4720 for 48 hours. Cell viability was measured by WST-1 assay. *p (vector vs wtAxl under PLX4720 treatment) = 0.024; *p (wt vs K567R Axl under PLX4720 treatment) = 0.007. **B.** EGFR transfected parental AM38 cells were treated with 0.1% DMSO, 5 uM PLX4720 or 1 uM HKI-272 for 48 hours. Cell viability was measured by WST-1 assay. *p (vector vs EGFR under PLX4720 treatment) = 0.004.

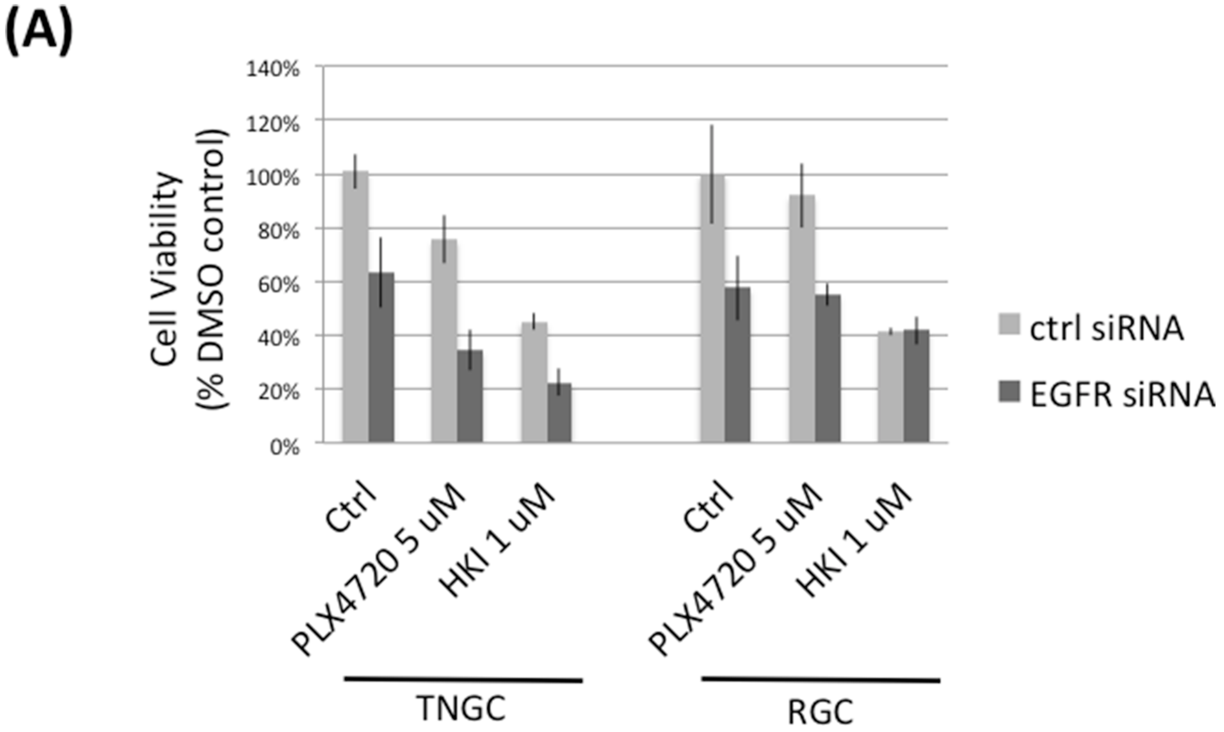

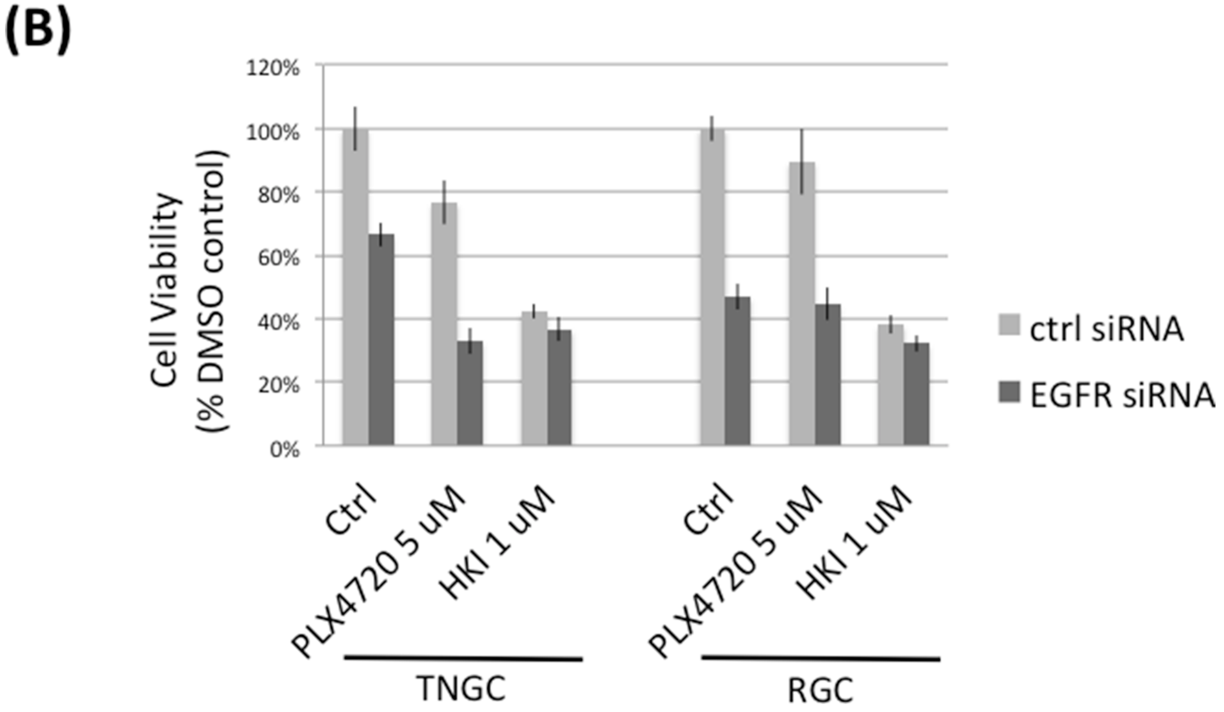

Supplementary Figure S6: AM38 A. and DBTRG-05MG B. RGCs and TNPCs were transfected with scramble control (light grey bars) or EGFR siRNA (dark grey bars) and treated with 0.1% DMSO, 5 uM PLX4720 or 1 uM R428 for 48 hours. Cell viability was measured by WST-1 assay.

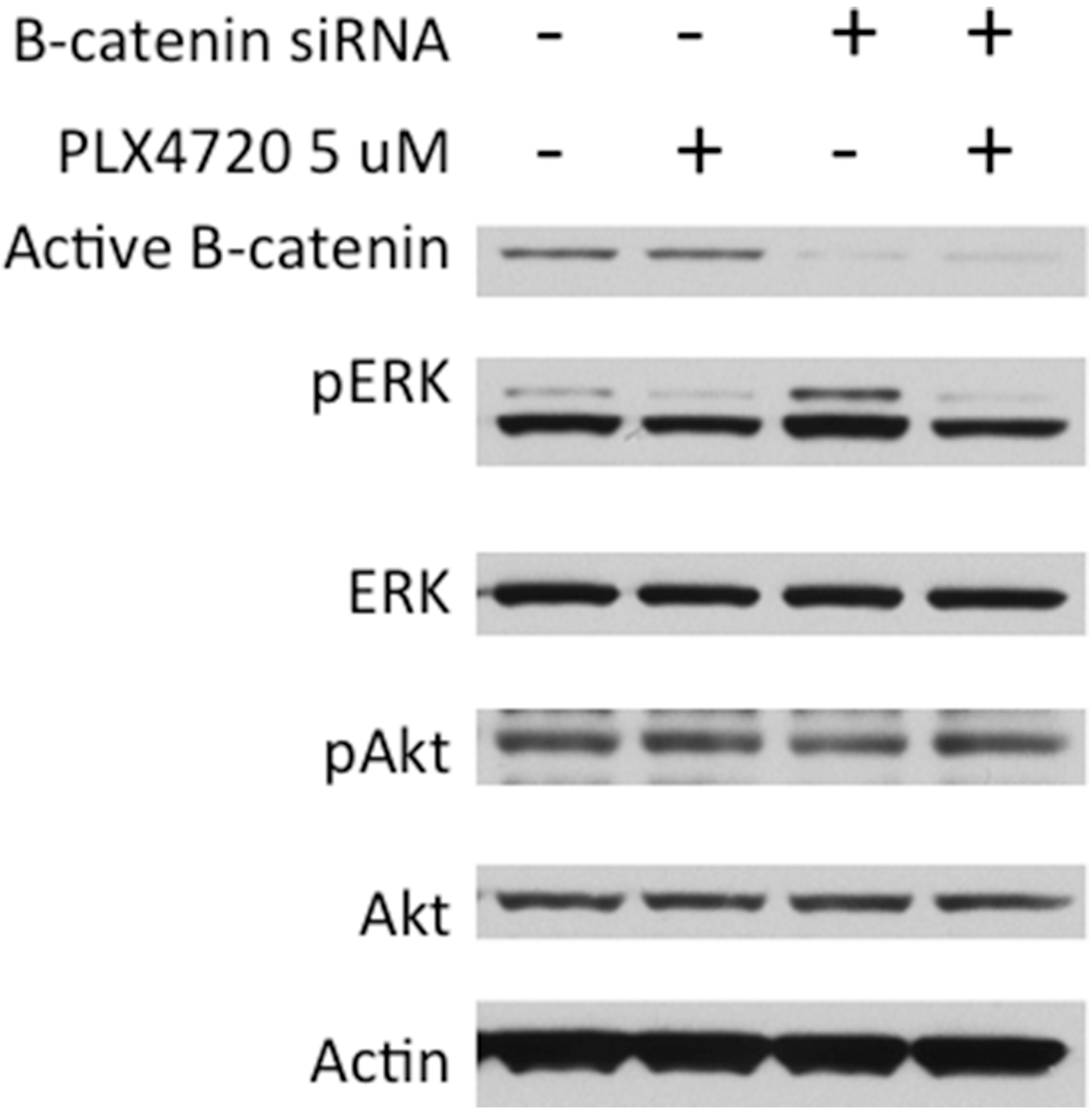

Supplementary Figure S7: DBTRG-05MG RGCs were transfected with scramble control or B-catenin siRNA for 48 hours. The transfected cells were then treated with 0 or 5 uM PLX4720 for 2 hours. Cells were then lysed and analyzed by immunoblotting.

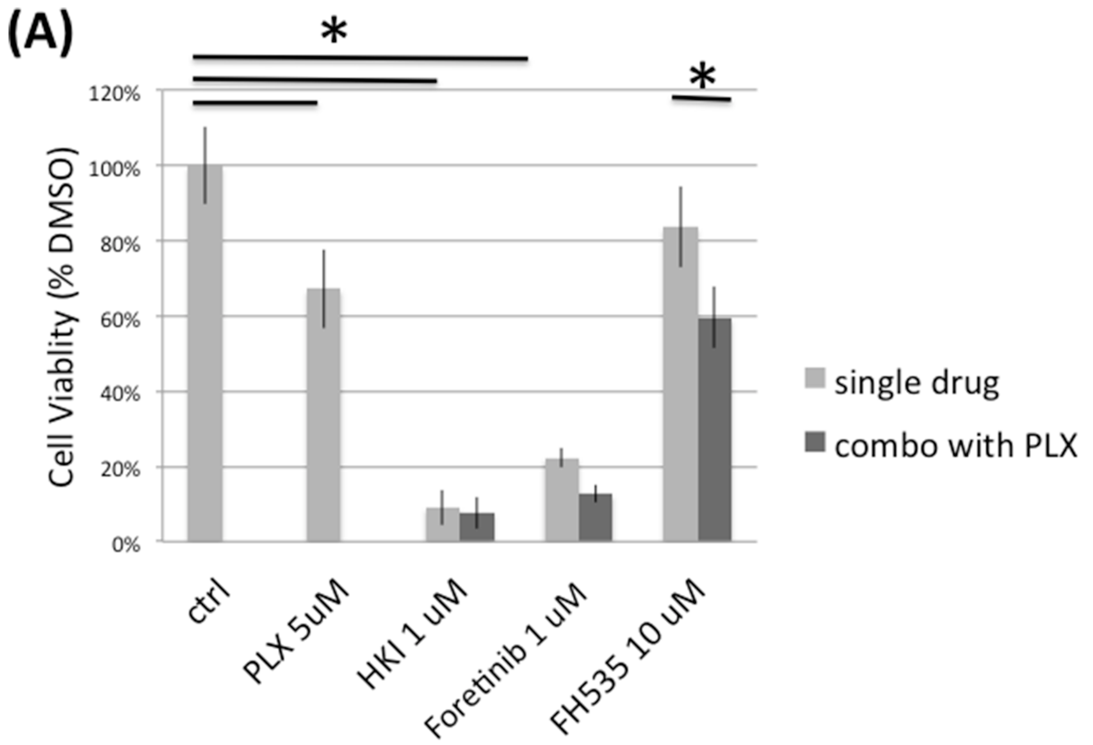

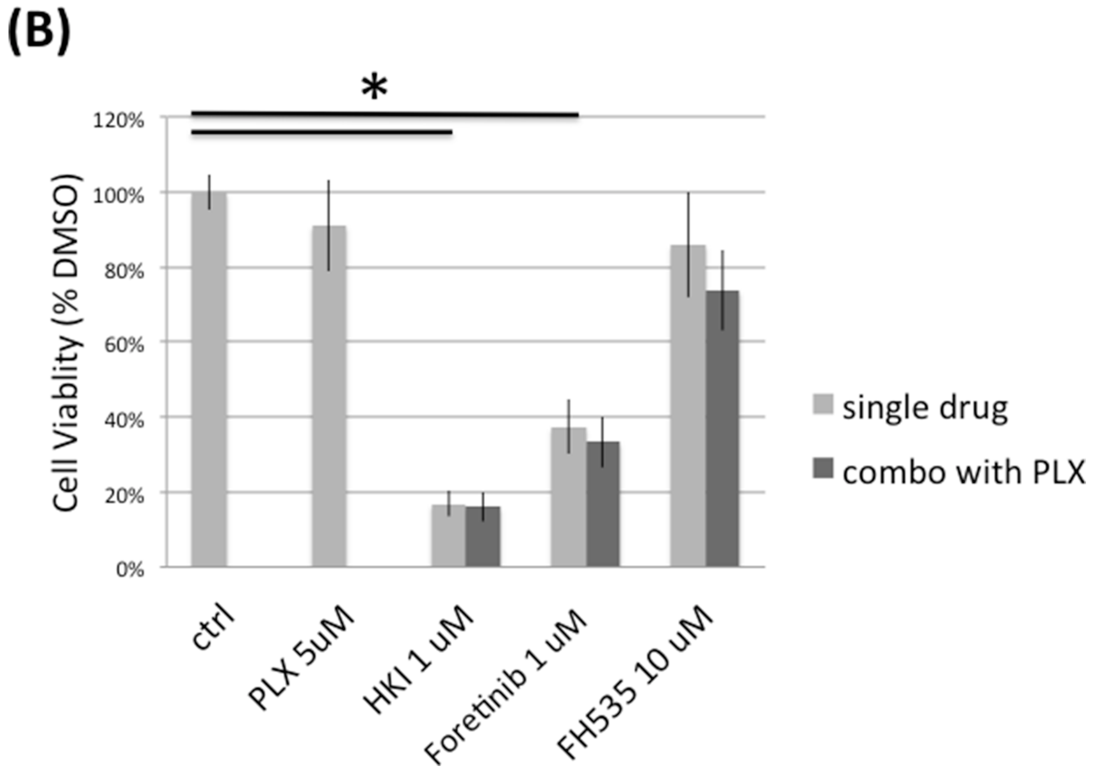

Supplementary Figure S8: AM38 TNPCs A. and RGCs B. were treated with single drugs (grey bars; 5 uM PLX4720, 1 uM HKI-272, 1uM foretinib, or 10 uM FH535), or in combination with 5uM PLX4720 (dark grey bars) for 48 hours. Cell viability was measured by WST-1 assay.

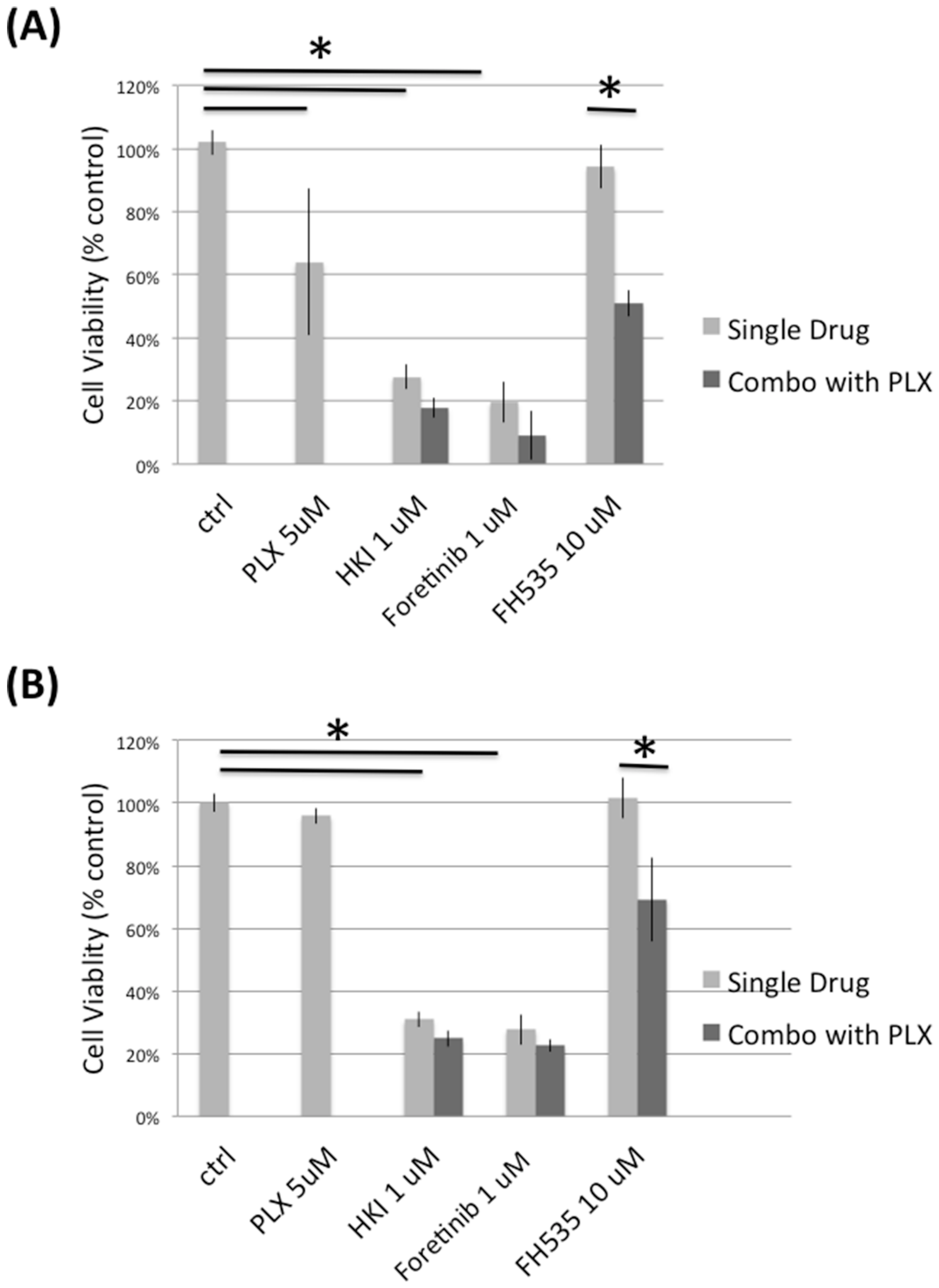

Supplementary Figure S9: DBTRG-05MG TNPCs A. and RGCs B. were treated with single drugs (grey bars; 5 uM PLX4720, 1 uM HKI-272, 1uM foretinib, or 10 uM FH535), or in combination with 5 uM PLX4720 (dark grey bars) for 48 hours. Cell viability was measured by WST-1 assay.
